# Three-dimensional-printed headcap with embedded microdrive system for customizable multi-region brain recordings with neural probes

**DOI:** 10.3389/fnins.2024.1478421

**Published:** 2024-10-17

**Authors:** Jeremiah P. Hartner, Dongyang Yi, Harrison L. Zhu, Brendon O. Watson, Lei Chen

**Affiliations:** ^1^Department of Psychiatry, University of Michigan, Ann Arbor, MI, United States; ^2^Department of Mechanical and Industrial Engineering, University of Massachusetts Lowell, Lowell, MA, United States

**Keywords:** headcap, microdrive, customization, multi-region electrophysiological recording, 3D printing, neural probes

## Abstract

Electrophysiological recordings from single neurons are crucial for understanding the complex functioning of the brain and for developing eventual therapeutic interventions. For electrophysiology, the accuracy and fidelity of invasive implantations of small devices remains unmatched. This study introduces an innovative, cost-efficient, 3D-printed headcap with embedded microdrive (THEM) system designed to streamline the manual labor-intensive *in-vivo* electrode implantation process for efficient and precise multi-region brain neural probe implantations. A custom bregma-referenced headcap design and fabrication, embedded microdrive integration, and upper support structure for probe packaging are described. With the Sprague Dawley rat as test species and medial prefrontal cortex and CA1 of the dorsal hippocampus as targets, surgeries and electrophysiological recordings were conducted to test the capability of the THEM system as compared to conventional surgical methods. By shifting manual stereotaxic alignment work to pre-surgical preparation of a fully assembled headcap system, incorporating fully preassembled upper support framework for packaging management, and easy customization for specific experiment designs and probe types, our system significantly reduces the surgical time, simplifies multi-implant procedures, and enhances procedural accuracy and repeatability. The THEM system demonstrates a significant improvement over conventional surgical implantation methods and offers a promising tool for future neuroscience research.

## 1 Introduction

Electrophysiological recordings of single-neuron activity are a pivotal methodology for deciphering the intricate mechanisms underlying brain function, and they facilitate a deeper understanding of the neural basis of behavior, cognition, motor coordination, as well as brain disorders (Jun et al., 2017; Allen et al., 2019; Durand et al., 2023). Despite the efforts towards large-scale non-invasive recording techniques (Min et al., 2010; Coyle et al., 2007; Hong et al., 2020; Mizuta and Sato, 2024) and the development of novel electrode materials and fabrication methods aimed at eliminating invasiveness (Hong and Lieber, 2019; Won et al., 2020), the pursuit of fast-timescale, high-fidelity, and wide-scale neural recordings necessitates electrode implantation strategies. Implantation-based recordings remain the gold standard for recording the activity of spiking brain networks *in vivo* (Frank et al., 2019; Stieglitz, 2020; Shen et al., 2023). Currently, silicon or flexible substrate-based neural probes with a high electrode site density are some best options for studying neuronal spiking activities essential for neuroscience research due to their resolution and reliability (Juska et al., 2023).

To determine how spiking activity correlates with behavior, chronic implantations of devices are performed so that animals can recover from surgery and behave naturally while brain activity is recorded (Vandecasteele et al., 2012). Recent innovations in chronic neural implantation have focused on improving the types of implants used: increasing local channel counts on each neural probe shank (1) or reducing tissue damage from implants (Viventi et al., 2011; Khodagholy et al., 2015). Thus, we continue to rely on implant-based recordings, but our surgeries are based on decades-old manual methodologies that do not take advantage of modern design and manufacturing capabilities.

Traditional implantation methods for such chronic recordings are characterized by a reliance on manual alignment using a stereotaxic device and temporary structural support during surgery, alongside other labor-intensive considerations like headstage construction. These surgeries require many hours of training and can also take hours to perform. They therefore act as a bottleneck on the use of high-tech implants, especially for multi-region brain recordings with multiple implantations needed.

To address this need, rapid advancements in digital design and 3D printing technologies have revolutionized the rapid prototyping of custom fixtures for *in vivo* rodent brain surgeries, offering low-cost tailored options for control and assistance during manual neural probe insertions (Bimbard et al., 2023; Juavinett et al., 2019; Luo et al., 2020; van Daal et al., 2021; Melin et al., 2023). Some of these new devices employ a novel 3D printed casing and rail device (Juavinett et al., 2019; Luo et al., 2020; Melin et al., 2023), or a payload-docking module for easier implantation/removal (Bimbard et al., 2023; van Daal et al., 2021; Vöröslakos et al., 2021). However, even though the 3D printed components, or metal drive components (Vöröslakos et al., 2021) remain lightweight in these designs, many of these devices extend the weight further from the head, and the bulky size of the system both limits the benefits of surgical efficiency/repeatability and prevents it from handling many probes or inserting probes at custom locations other than the fixed-gap back-to-back configuration determined by the fixture design. Even in examples where these rail or payload-docking systems were employed to potentially implant 6 probes, the system was not well-suited for handling the packaging and output connectors, and due to the compromised stability and stress on the probes, the system was unlikely to be feasible for recording in freely behaving animals (Melin et al., 2023). Additionally, all these alternative implantation systems still require practices that are otherwise identical to more traditional stereotaxic implantation and cement fixation methods, requiring great care and surgical time to implant each probe iteratively.

Each of the 3D-printed systems detailed above were designed to augment traditional stereotactic procedures and have shown promise in enhancing the base stability and accuracy of insertion depth adjustments through microdrive mechanisms. Despite these innovations, facilitating a degree of surgical efficiency, the complexity and demand of the surgical process remain significant challenges. Limitations arise particularly in adapting these systems to accommodate large numbers of probes in a manner that improves needs for surgical skill and time. Additionally, the stereotactic frame remains as the foundation of each of these approaches. Due to the iterative nature of the stereotaxic-based insertion, difficult surgical maneuvers must be performed repeatedly, each taking significant time. Each probe must be carefully mounted to the stereotaxic arm and guided to the insertion site, followed by anchoring and untethering of the probe apparatus before the method is repeated for each probe. Furthermore, they must be done in increasingly small spaces as the number of probes increases, which risks damage to both the probe being inserted and the probes previously mounted to the head. Finally, each of these previous approaches has significant difficulty in managing the probe packaging atop the head during surgery. Because of these challenges, other complications arise including increased likelihood of animal health complications, increased surgeon exhaustion and error, and complex and difficult to replicate surgeries. Therefore, speeding up and simplifying the surgical process by shifting away from a stereotaxic-based iterative insertion process is a major goal.

**Figure 1:**
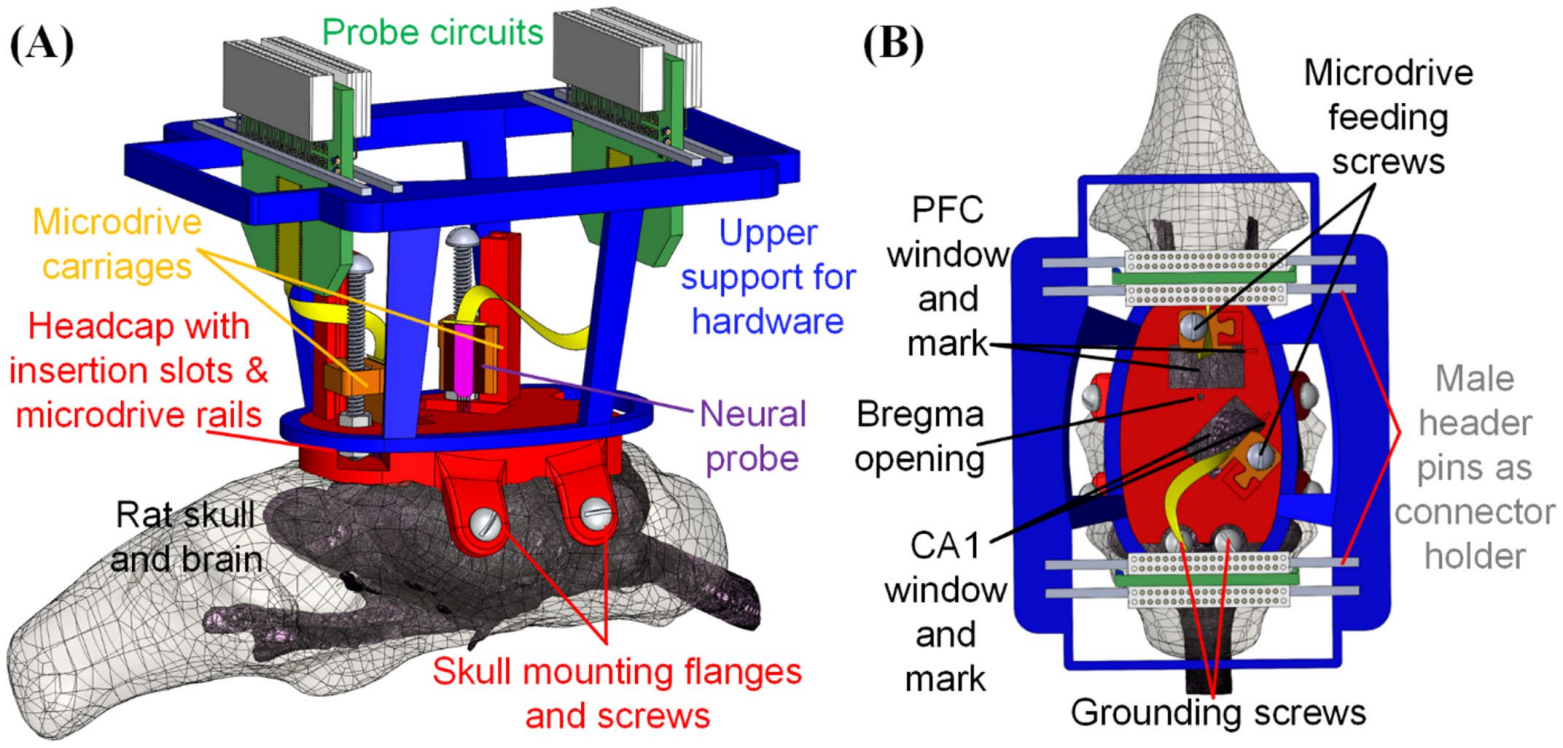
Design of fully assembled THEM system for multi-probe implantations: **(A)** overview and **(B)** top view of key components and features. This is a particular example design, but many others can be made via computer-aided design (CAD)—to allow targeting any brain location or using different devices.

This study introduces our low-cost and customizable three-dimensional-printed headcap with embedded microdrive (THEM) system to refine and streamline the surgical implantation process for multiple electrodes and/or neural probes. The THEM implantation system is composed of (1) a custom designed 3D printed headcap for pre-surgery bregma-reference insertion location determination (Yi et al., 2022), (2) embedded microdrives made of 3D printed parts and standardized fasteners for stereotax-free implantation/recovery, and (3) upper support structure for managing the probe back-end circuits and connectors, as shown in Figure 1. This approach enables precise pre-surgical planning and preparation, with custom probe insertion sites predetermined, digitally designed, and 3D printed based on accurate bregma coordinate mapping (Yi et al., 2022). In addition, the THEM system allows each neural probe to be affixed to the corresponding embedded microdrive in the headcap prior to surgery, so upon head cap installation, each probe is already aligned at the insertion position. It can then be implanted to the desired depth by drive screw turning only—without a stereotaxic arm. As the neural probes are costly and fragile and their positioning and anchoring are the most intricate and time-consuming portions of current manual procedures, the THEM system could shift most work to a pre-surgical time not limited by anesthesia or other surgical constraints, resulting in a marked improvement in surgical efficiency. This manuscript elaborates the design, fabrication, surgical procedures, and recording results utilizing the THEM system through a proof-of-concept study implanting two silicon probes to the prefrontal cortex (PFC) and hippocampal subfield CA1 of Sprague–Dawley rats.

## 2 Materials and methods

In this section, we detail the design and fabrication of our custom THEM system, including the headcap with bregma-reference insertion slots, embedded microdrives for neural probe feeding, and the upper support structure for back-end circuits and connectors. The system’s evolution from a single-site surgical platform to a multi-region, custom-built microdrive-based insertion mechanism is illustrated by iterative designs and detailed through test trials of each component of the THEM system individually.

### 2.1 Custom headcap with bregma referenced insertion sites

**Figure 2:**
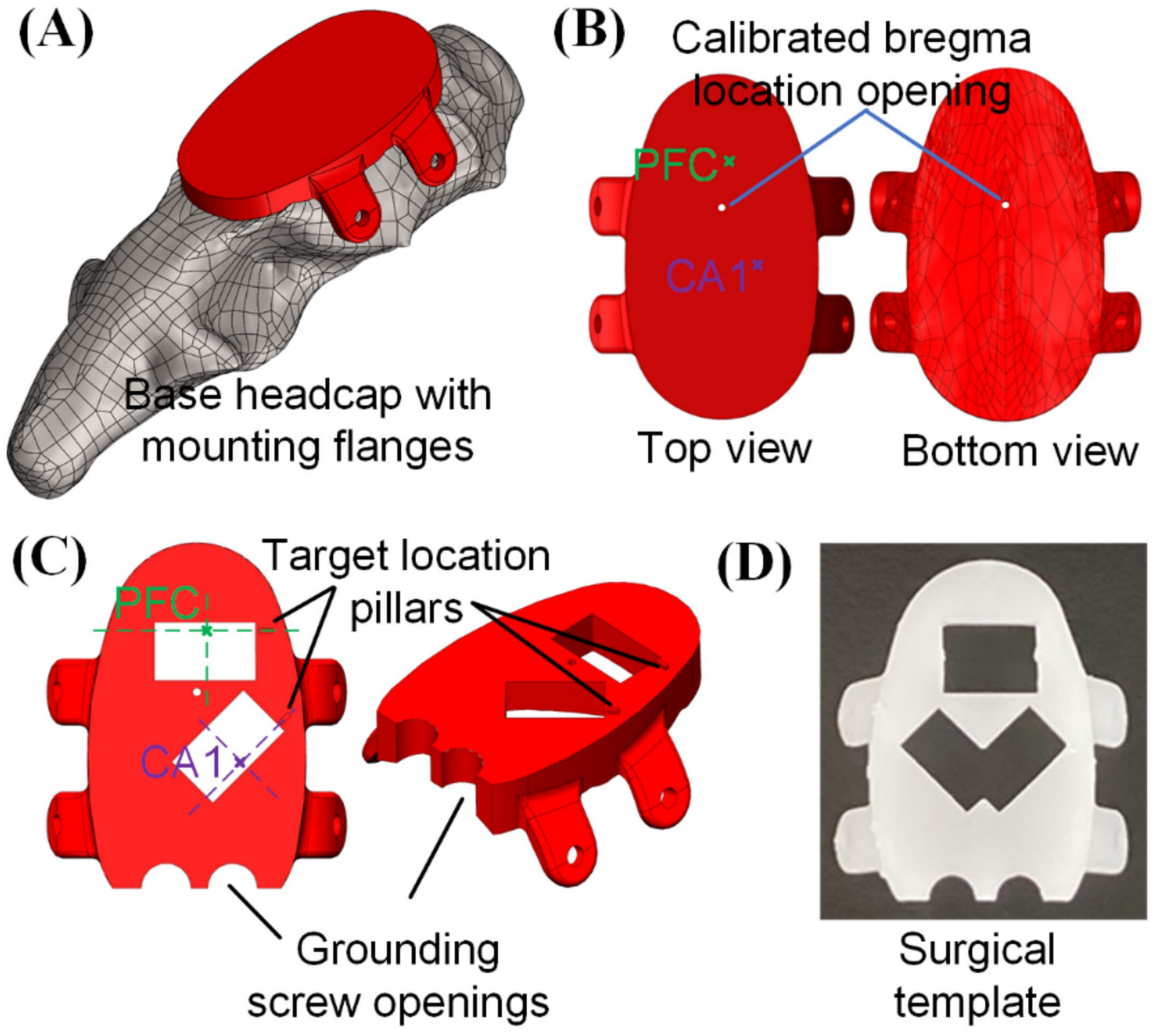
3D printed head cap with bregma-referenced custom insertion locations: **(A)** base headcap based on CT scan of rat skull, **(B)** bregma location calibrated based on multiple animals (*n* = 6) and bregma-referenced custom insertion location identification, **(C)** creation of implantation windows and grounding screw openings on the cap, and **(D)** usage of such cap as a surgical preparation template for skull openings (picture shows a 3D-printed template capable of doing both left and right CA1).

In our previous work, we developed a 3D-printed headcap derived from computerized tomography (CT) of a Sprague–Dawley rat skull (Yi et al., 2022), as in Figure 2A, with the bottom of the cap matching the natural skull underside. The headcap design based on single rat CT scan was shown to conform well with multiple Sprague–Dawley rats of various sizes via surgical testing. In that work, we reported that over multiple animals, estimated bregma locations centered around manually verified bregma with minimal bregma alignment error [<1.0 mm along the anterior-posterior (A/P) axis and <0.3 mm along the medio-lateral (M/L) axis], as in Figure 2B (Yi et al., 2022). As a result, all elements of the 3D printed design can be centered on a particular location in the headcap known to match bregma and brain coordinates (A/P and M/L) of the desired insertion sites could be integrated into the headcap design. At the time of surgery, the surgeon could physically “click” the 3D printed cap onto the animal’s head at a particular physical catch point in the cap-skull interface (essentially slide the cap forward until it stops) and perform craniotomy and probe implantation based on the printed openings that can serve as “craniotomies.” This eliminates the need for individual bregma measurement or calibration. Though this method of attaching the headcap is repeatably accurate for aligning headcap and skull bregma, a viewing window can be created at headcap bregma for surgeon’s confirmation of bregma point alignment during surgical installation of the cap. We use this same skullcap here, but with adapted design elements for multiple silicon probes.

The procedure to integrate desired insertion site coordinates into the headcap design is elaborated below, with the targeted PFC and CA1 in this study as an example. Based on the rat brain atlas (Paxinos and Watson, 2006), center coordinates of PFC and CA1 regions from bregma were, respectively (anterior/posterior, medial/lateral, dorsal/ventral in the unit of mm): (3.2, 0.5, −3.0) and (−3.8, 2.4, −2.0), which could be identified on the base headcap design, as highlighted in Figure 2B. Based on these coordinates, craniotomy opening locations were printed into the cap and a 6.1 mm by 3.5 mm rectangular opening was used in this study for each insertion location to accommodate for the neural probe width. A small location identification pillar was added to the design along the shorter edge of the rectangular opening and the intersection between its extension line and the rectangular opening center line would be the targeted insertion location of PFC and CA1, as shown in Figure 2C. Such a cap serves as the base template for embedded microdrive design and integration to be elaborated in section 2.2. Besides that, with two grounding screw openings added, this cap could also be used as a surgical template to create craniotomy openings, pre-install grounding screws, and pre-drill headcap mounting holes on the sides of the skull following the flange holes, as shown in Figure 2D. Custom headcap designs were created using CAD software SolidWorks (Dassault Systèmes, Vélizy-Villacoublay, France). All 3D printed parts in this study were fabricated with a stereolithography 3D printer (Form 3B by FormLabs, Somerville, MA) with liquid resin (Clear Resin V4 by FormLabs, Somerville, MA). After printing, the components were thoroughly cleaned in 99% isopropyl alcohol (Form Wash by FormLabs, Sommerville, MA) and then cured under ultraviolet light (Form Cure by FormLabs, Sommerville, MA) to enhance their rigidity for assembly and surgical use.

### 2.2 Integration of embedded microdrives

**Figure 3:**
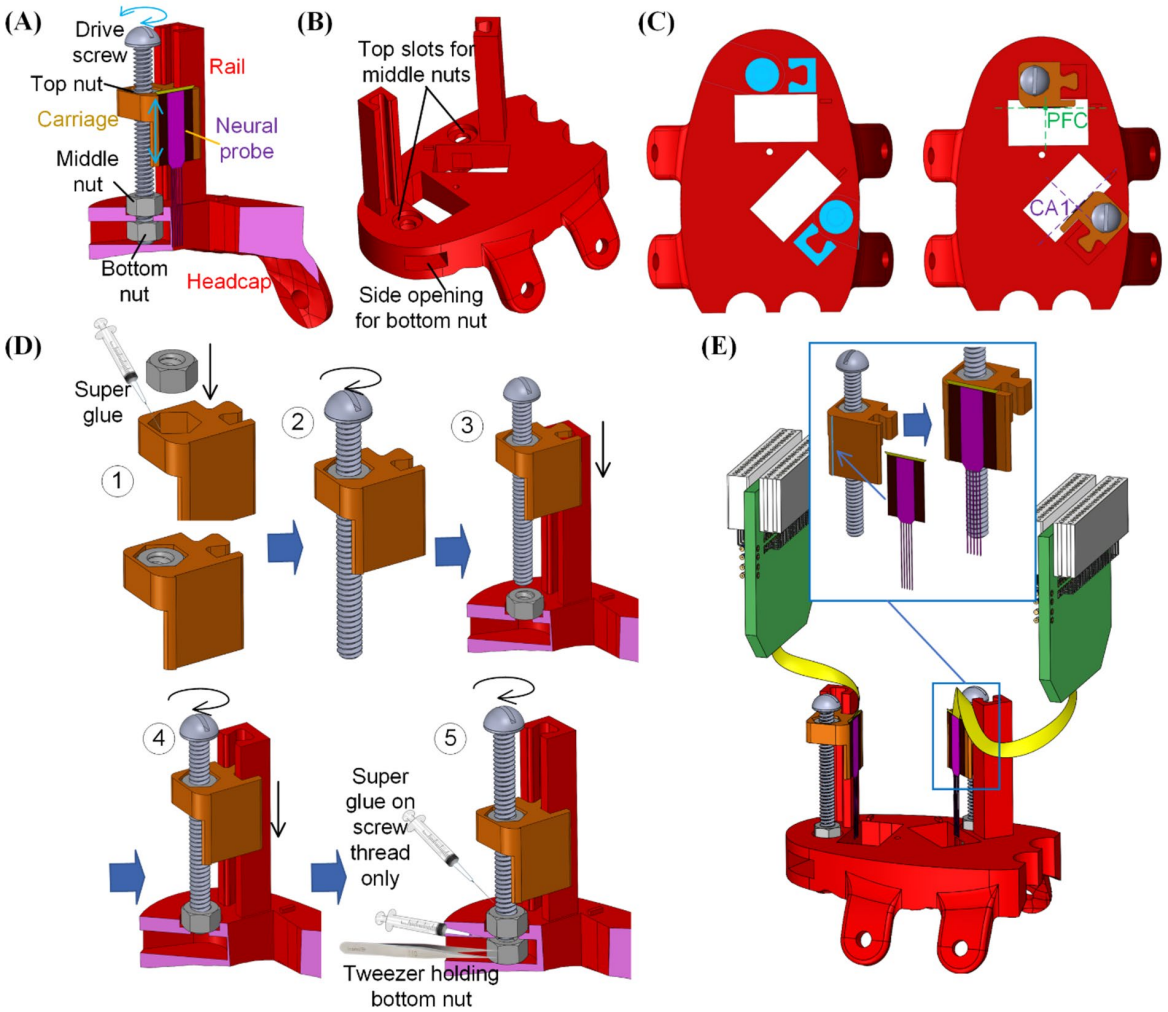
Integration of the embedded microdrives into the 3D printed headcap with custom insertion locations: **(A)** overview of the microdrive functioning mechanism, **(B)** modification of the 3D printed headcap for microdrive integration, **(C)** positioning of the microdrive features for neural probe alignment, **(D)** assembly steps for the embedded microdrives, and **(E)** attachment of neural probes onto the assembled microdrive carriages.

We next integrated microdrives into the heap cap design to form the THEM system for controlling the dorsal/ventral (D/V) axis insertion depth for multiple implants. The working principle for the proposed embedded microdrives is shown in Figure 3A. Each microdrive was composed of (1) one rail integrated and printed together with the head cap as one piece for maximum strength and stability, (2) one 00-90 ½ inch long brass screw, (3) three 00-90 sized nuts and (4) one 3D printed carriage housing a nut inside and matching the rail geometry for D/V sliding. The bottom two nuts were glued to and rotated together with the screw, which held the 00-90 drive screw axis to the headcap during rotation. The third top nut was glued inside the carriage and served as the moving component in the lead screw structure to linearly feed and/or retract the neural probe precisely during drive screw rotation as shown in Figure 3B. Based on the 00-90 screw pitch, each full turn of the screw would lead to 282 μm insertion/retraction depth of the neural probe.

Next to each craniotomy window on the dorsal surface of the headcap, a rail was added as the insertion guideway and a counter hole was implemented for positioning the middle nut. Underneath the headcap surface, an opening was created from the side of the cap to house the bottom nut. As shown in Figure 3C, the position of such rails and nut housings were determined so that upon assembly of the carriage and attachment of the neural probe, the center of each probe was placed above the targeted region.

After 3D printing of (1) the designed headcap with embedded guide rails as one piece and (2) probe carriages for each insertion site following methods as elaborated in section 2.1, assembly of the THEM system is as shown in Figure 3D following five steps:

Step #1: Apply instant super glue (High Strength All Purpose Super Glue by Gorilla Glue Company, Cincinnati, OH) within the nut holder in the carriage and firmly fix the top nut into it.

Step #2: Rotate the drive screw into the carriage-top nut assembly to engage the drive screw and top nut.

Step #3: Place the middle nut into its slot on top of the headcap, then slide the assembled screw-carriage into the corresponding headcap rail.

Step #4: Rotate the drive screw until its thread pass through the bottom surface of the middle nut. The carriage would be lowered during this process.

Step #5: Use a pair of tweezers to place the bottom nut into the cap side opening and align it with the drive screw. Rotate the drive screw to engage it with the bottom nut. Before tightening the bottom two nuts into the final position, apply a small amount of super glue along the thread of the drive screw (not between nut and cap surfaces). After further turning the screw into the final position, the super glue would fix the relative angular position between the bottom two nuts and the drive screw. Thus, during the lead screw motion, the bottom nuts would hold the drive screw in position while rotating with it.

After full assembly of all microdrives, the rail-carriage interfaces were coated with Vaseline to lubricate a smooth feeding motion. Neural probe implants were then mounted to corresponding carriages. To ensure accurate mounting position of the neural probes onto the carriage, a small ledge was created on the edge of the carriage mounting surface, as shown in Figure 3E. When adhering the neural probe to the corresponding microdrive, the neural probe would have its back surface laid flat against the mounting surface while its edge was pushed against the step as a guide to ensure accurate alignment along all directions. Mounting was done using a drop of cyanoacrylate on the back of the plate from which the shanks emerge (Figure 3E). We used multiple types of silicon probes in our studies, including A1x64-Edge-6 mm-22.5-177-H64LP_30mm and Buzsaki-5x12-H64LP_30mm by NeuroNexus, Ann Arbor, MI and P64-4 by Diagnostic Biochips, Glen Burnie, MD, to ensure flexibility of our probe interface to accommodate various probe designs. The mounting surface was designed to be offset from the insertion opening on the base headcap to avoid any undesired contact during insertion. The flat mounting surface was also built with an extension downward into the opening to gain additional insertion depth by compensating for the dead space between the headcap bottom and brain surface as well as the thickness of the headcap. This embedded multi-microdrive system enabled pre-assembly of the main head stage with all neural probes mounted beforehand. It thereby eliminated the handling and alignment of individual external drives during surgery.

### 2.3 Upper support structure for back-end circuits and connectors

**Figure 4:**
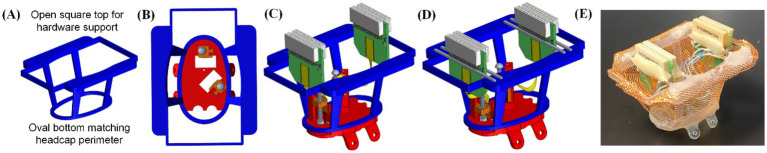
Upper support structure for back-end circuits and connectors: **(A)** overview of the upper support design, **(B)** fitting between the upper support and headcap base, and assembly of the upper structure including **(C)** aligning the upper support with headcap outer perimeter and **(D)** cementing the back-end electronics to the support structure through male header pins, and **(E)** picture of fully pre-assembled headcap ready for implantation, including a copper mesh wrap.

For multi-neural probe implantations, handling and temporary fixation of the back-end electronics can be tedious and time-consuming, especially within the tight space above the animal’s skull, as shown in Figure 3E. To address this issue, an upper support structure was designed as in Figure 4A and fabricated as a separate 3D printed fixture. The upper support structure was composed of an oval bottom shape matching the outer perimeter geometry of the headcap base, as in Figure 4B. Thus, after probe fixation onto the carriages as in Figure 3E, the upper support structure could be slid onto the headcap and cemented in place (Figure 4C). Then, each of the amplifiers/connectors could be cemented to one or two support rails (made of male header pins in this study) along the top of the frame, positioning the connectors in an orderly fashion while maintaining access to the drive screws for peri- and post-surgical depth adjustments (Figure 4D). The upper frame could then be wrapped in copper mesh to act as a mini-faraday and grounding attachment point for the probe (Figure 4E). At this point, the entire THEM system-based headstage was fully assembled before surgery, allowing the benefit of completing the intricate work in a non-surgical environment rather than on top of an anesthetized animal’s head during a difficult hours-long surgical procedure.

The total weight of the full assembly used for the dual probe proof-of-concept trial elaborated in this study was 7 g (interface plate with two integrated drives = 1 g, upper housing = 1 g, copper mesh Faraday cemented to upper housing = 2 g, two NeuroNexus probes = 3 g). To create a more rigid final structure, the copper mesh can be reinforced/encased with dental acrylic, resulting in a final weight of 10 g. Given that typical rat headcaps designed for silicon probe implants weigh 10–20 g (Vöröslakos et al., 2021; Headley et al., 2015), the THEM system is a lightweight design ideal for long-term electrophysiological recordings.

### 2.4 Animal surgery procedure and recording experiment design

The animal procedures were conducted following the guidelines set by the Institutional Animal Care and Use Committee (IACUC) of the University of Michigan. Animals used in this study were Sprague Dawley rats aged between 3 to 6 months, sourced from Charles River Laboratories, Wilmington, MA. All animals were maintained in an enriched living environment under a standard light-dark cycle (lights on from 6 a.m. to 6 p.m.) and had unrestricted access to food and water. Before the surgery, the rats were anesthetized using isoflurane, placed in a stereotaxic apparatus, and administered subcutaneous carprofen (5 mg/kg) for analgesia and intraperitoneal methylprednisolone (30 mg/kg) to minimize brain swelling. A local anesthetic, bupivacaine (1 mg/kg), was given subcutaneously to the scalp before surgical incision. A sterile scalpel blade was used to create an incision along the skull’s midline, exposing the bone.

Various forms of the headcap were trialed during the development process, including (1) the bare headcap without microdrives or with commercially available metal microdrive, (2) the 3D printed headcap with our embedded microdrive(s), and (3) fully pre-assembled THEM system including microdrive-mounted silicon probes supported by the upper housing structure. For all headcap versions trialed, a template cap as in Figure 2D was printed at the same time as the components of the headcap to be used as an alignment and cranial window guide prior to anchoring of the permanent headcap. The template cap is a duplicate of the final headcap used during assembly, but lacking pillars for microdrives. The template cap can also include a hole at the universal bregma point to be used as a viewing window to ensure the cap is aligned to the animal’s true bregma point, though in our previous trials we found this viewing window is not necessary to achieve accurate probe targeting.

**Figure 5:**
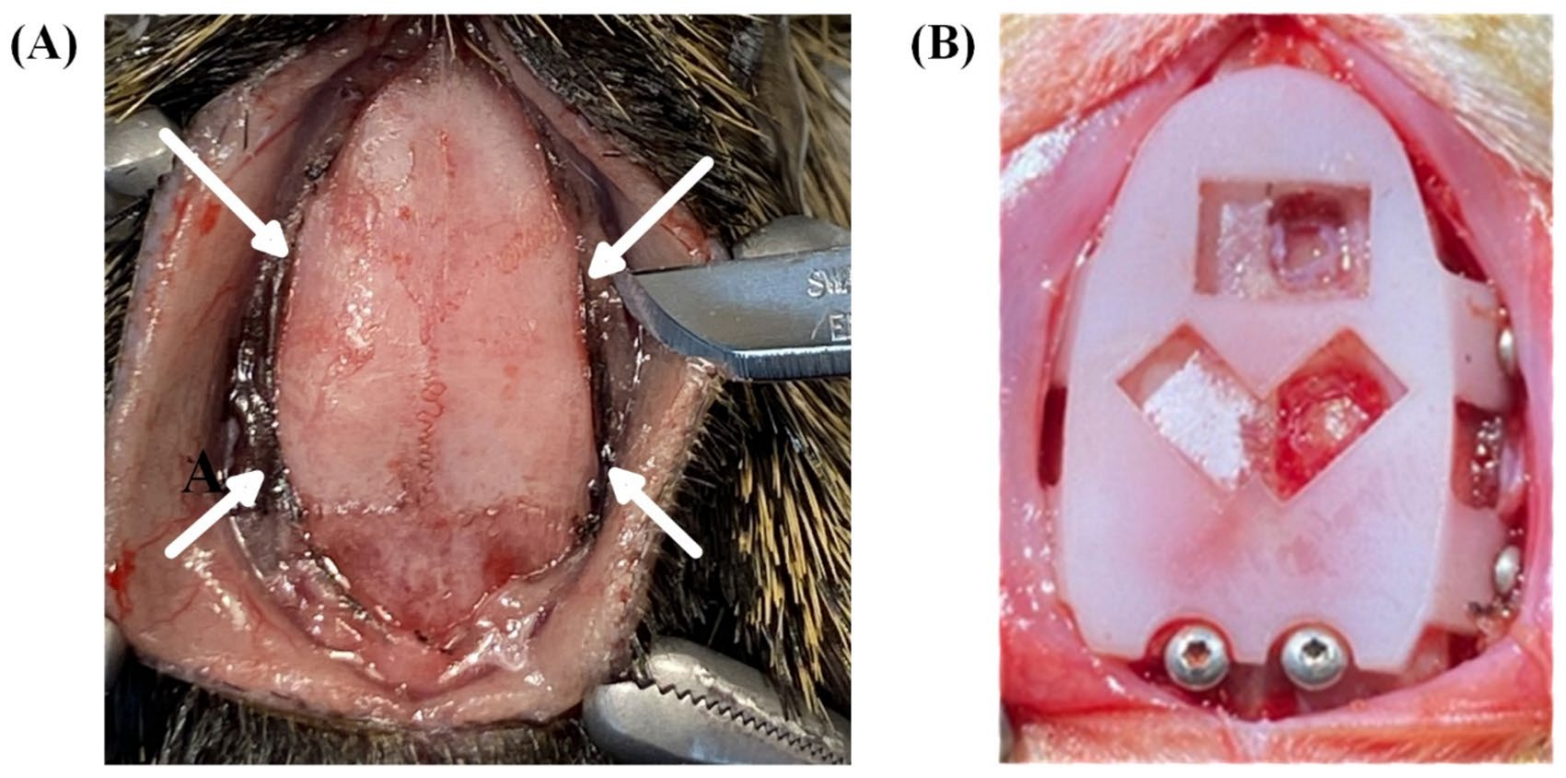
Surgical procedures and headcap placement. **(A)** Surgical preparation for headcap fixation flaps to be positioned under the muscle along the temporal bone. Arrows indicate incision sites to create channels under the muscle tissue immediately lateral to the temporal ridge. **(B)** Placement of template cap used to predrill fixation and grounding screw holes and to trace bregma-referenced cranial window locations.

For the headcap to achieve optimal fit on the dorsal surface of the skull, the fixation flaps on the sides of the cap were positioned underneath the fascia/muscles on the side of the skull. To achieve this, a scalpel was used to make small cuts in the fascia immediately lateral to the temporal ridge (Figure 5A). The cuts were large enough to accommodate the sliding of each flap along the temporal bone under the fascia, taking care to preserve as much muscle attachment as possible. The cap was aligned to bregma either using the bregma viewing window or sliding the cap along the skull until the cap sat flush. Once aligned, we used the template cap as the craniotomy template. First, we re-drilled holes for the side screws using a dental drill (MH-170 by Foredom, Bethel, CT, United States) with a 0.9 mm diameter burr. Next, we traced the locations of the cranial windows using a 0.5 mm diameter burr along the window edge, thereby eliminating the need for stereotactic measurements for probe positioning (Figure 5B). Once the anchor points and cranial window locations are drilled, the template cap was removed. Two stainless steel screws (00-90 thread with 3/32″ length) were inserted through the skull above the cerebellum to function as ground and reference points. Then full craniotomy windows were created on the skull along the cranial window marks, followed by durotomy of the exposed regions. Sterile saline was periodically added to each craniotomy window to avoid drying of the membranes/tissue.

After the preparation of all implantation sites, the permanent headcap was placed on top of the skull, clicking back into the same placement as before. Each probe shank was raised above the cap window to avoid shank damage during cap anchoring. Then each of the side fixation flaps was aligned along the corresponding pre-drilled holes. The headcap was attached to the sides of the skull using four fixation screws (00-90 thread with 1/8″ length) through the headcap fixation flaps. To reinforce stability, the cap was cemented with dental acrylic (UNIFAST Trad by GC America Inc., Alsip, IL, United States) along the perimeter of the headcap-skull junction, creating a long-lasting anchor for chronic recordings.

Once the cap was anchored to the skull, each drive screw was turned to achieve desired probe insertion depth. Depth calibration could be achieved in multiple ways, though the easiest and most reliable way was to count screw turns (282 μm insertion depth per full turn) from the point of initial contact with the brain. To visualize the probe’s initial contact and penetration, the cranial windows on the interface plate were designed to be larger than probe-fit alone. If smaller cranial windows were desired for high-density implantations or visualization of the insertion was not possible, the depth of the probe could also be calibrated by moving the probe flush to the bottom of the cap prior to surgery to learn the drive position corresponding with headcap bottom. Probes should then be retracted for safety with turns counted. After the headcap was anchored, the probes could be returned to the zeroed position ensuring the probe depth flush with the cap bottom. To account for any dead space between the cap and the brain, an estimate of the skull thickness could be made depending on the insertion coordinates. For our purposes, we used large headcap windows to visualize insertions, but we also found that skull thickness was approximately 1 mm for all our implant locations.

After a desired probe depth was achieved, the craniotomy windows were filled with a mixture of equal parts mineral oil and paraffin wax to protect the probe and brain for chronic recordings. Due to the difficult and time-consuming process of fabricating hardware housing and organizing output connectors, especially while working in tight spaces around multiple fragile probes, we highly recommend pre-assembly of the full THEM-based headcap system including the 3D-printed frame, as detailed above. After surgery, animals were given 1 week to recover prior to recording, and regularly monitored for signs of pain, distress, and infection.

### 2.5 Electrophysiological recording

Electrophysiological recordings were performed using 64-channel headstages (RHD2132 by Intan Technologies, Los Angeles, CA) connected to a USB interface board (RHD2000 by Intan Technologies, Los Angeles, CA) and sampled at 20 kHz. After a one-week post-surgery recovery period, animals were recorded in their home cage once per week for the first week, and biweekly for up to 4 months. Each recording lasted 2–6 h during the lights-on period.

## 3 Results

To test the feasibility of the THEM system, we inserted a total of 20 silicon probes in 12 different rats through a series of iterative and increasingly complex implantation surgeries. Surgical trials for probe implantations included: direct insertion of probes through the base headcap without conventional stereotaxic drives, insertion of probes through the base headcap while attached to commercially available metal microdrives, and insertion of probes mounted to our THEM microdrives either with or without a pre-assembled upper frame. We used the medial prefrontal cortex (*n* = 4) and CA1 of the dorsal hippocampus (*n* = 8) as recording targets.

### 3.1 Testing of the base headcap

**Figure 6:**
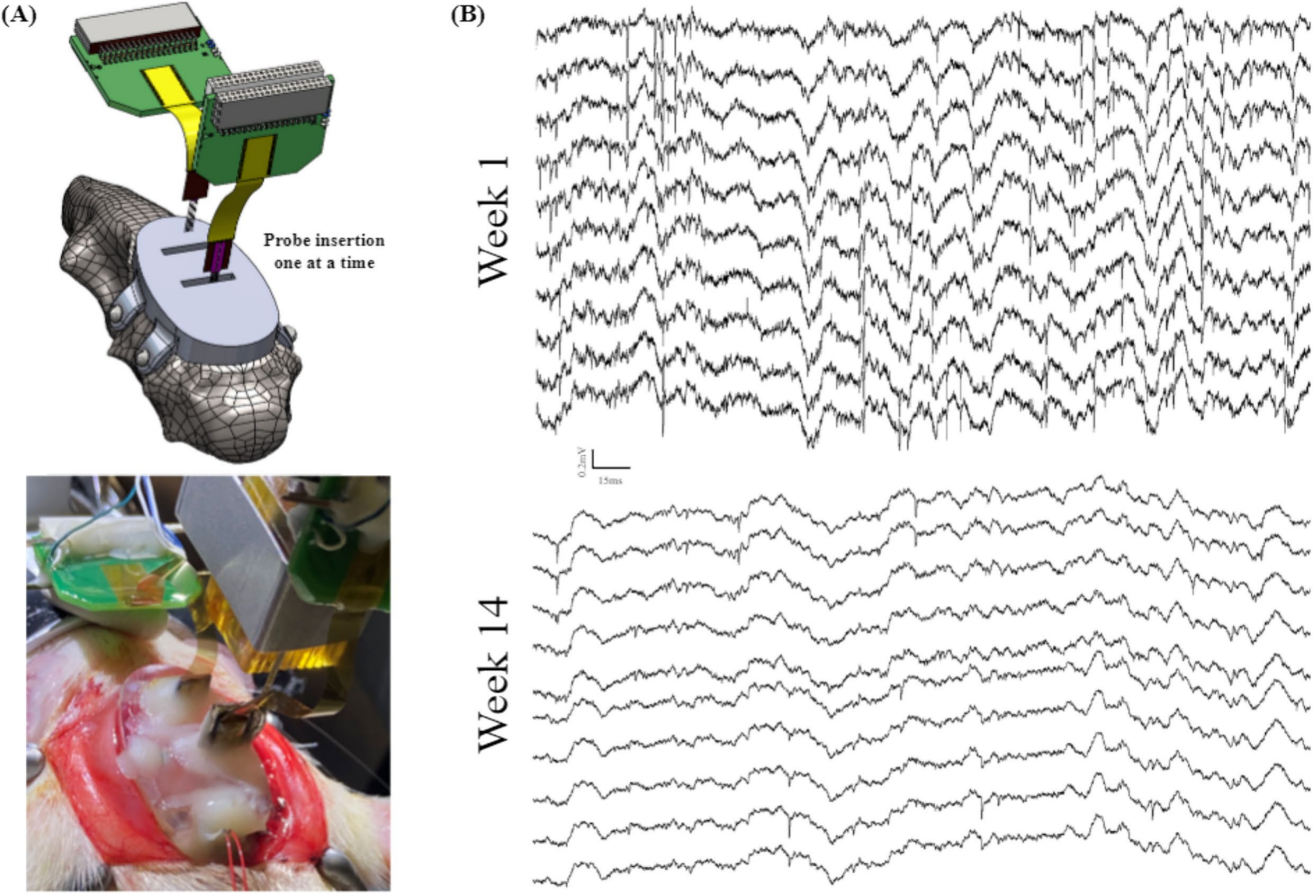
Animal recordings to validate the performance of a base headcap: **(A)** schematic view (top) and surgical picture (bottom) of the mPFC and CA1 probe insertions through the 3D printed headcap without microdrives and **(B)** electrophysiological recordings from (A) highlighting LFP and spiking activity in a subset of traces over the course of 14 weeks.

To ensure the targeting accuracy and feasibility of our headcap-based implantation system, we started by inserting silicon probes directly through the base headcap in a single animal (Figure 6A). This trial was performed as proof-of-concept to establish that the headcap could target brain regions of interest without measuring stereotaxic coordinates for each probe. Instead, we achieve accurate targeting by simply lowering the probe through the center of the bregma-referenced headcap windows. Using this method, we successfully recorded from the right PFC and CA1 for 14 weeks. Recordings demonstrated normal local field potential (LFP) signal, as well as spiking for the entire duration of recording, as shown in Figure 6B, indicating the 3D printed headcap does not interfere with traditional probe insertion methods and/or electrophysiological recording protocols. We did observe drift in spike amplitudes over time, which can be seen with the lower amplitude spikes observed at week 14, as shown in the bottom of Figure 6B.

### 3.2 Testing of headcap-based insertion using microdrives

**Figure 7:**
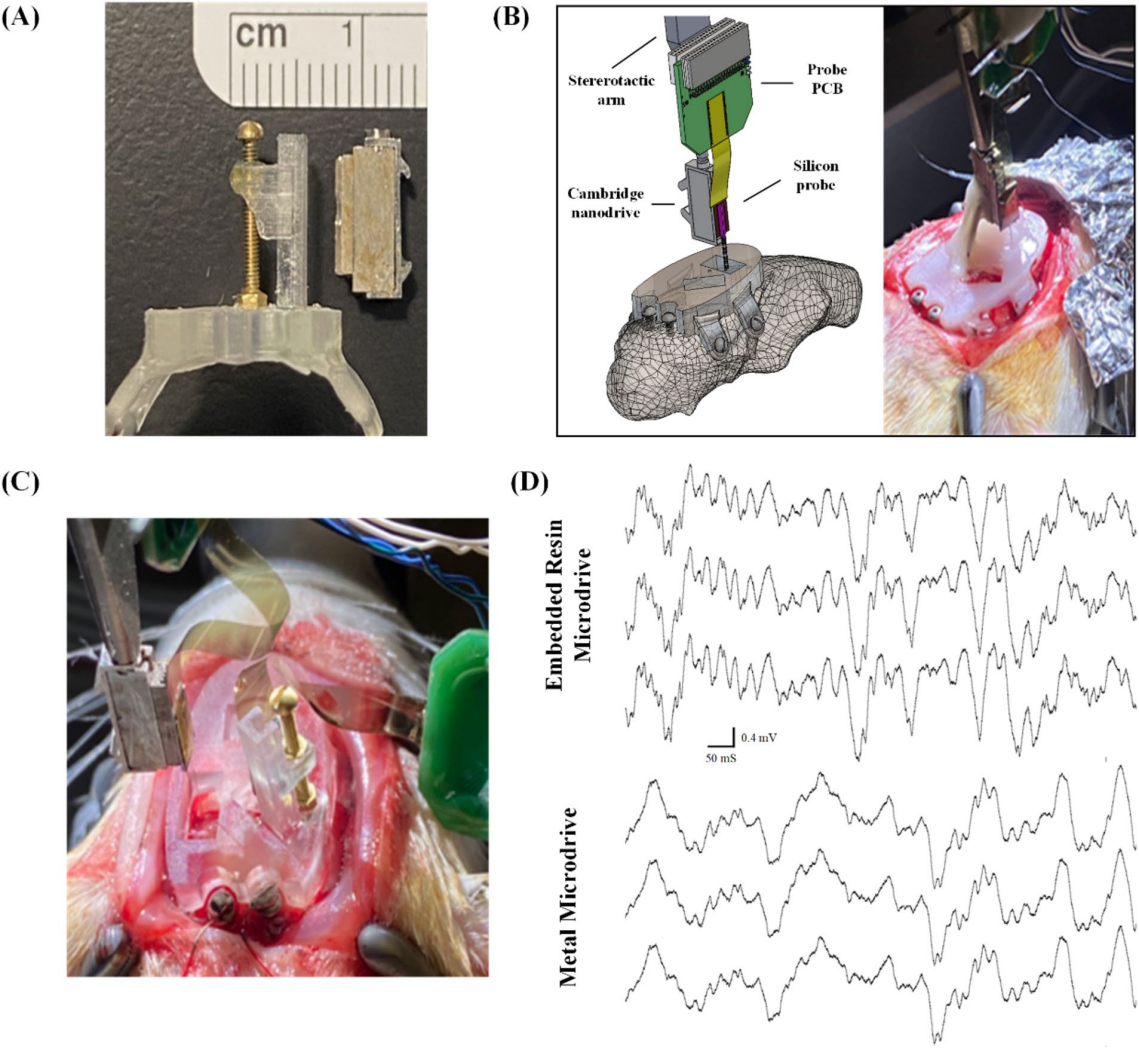
Surgical testing and validation of the embedded microdrives: **(A)** side-by-side comparison of Cambridge nano-drive (right) and our embedded 3D-printed and assembled microdrive, both measuring 11 mm × 5 mm × 2.5 mm; **(B)** schematic view and surgical picture of single probe insertion using commercial metal microdrive through the 3D printed headcap as a baseline experiment, **(C)** surgical picture of the comparison experiment inserting two probes: one with commercial metal microdrive and another with our embedded microdrive, and **(D)** electrophysiological recordings highlighting LFP delta and spindle signals from a subset of CA1 channels from probes attached to both the embedded resin microdrive (top) and commercially available metal microdrive (bottom).

In this section of experimental study, we tested commercially available metal microdrives of similar footprint (Figure 7A) as the baseline to evaluate our 3D printed embedded microdrives. In a single animal, we inserted 1 probe into right mPFC and 1 probe into right CA1, both mounted to metal micro-drives (Nano-Drive by Cambridge NeuroTech, Cambridge, United Kingdom) as shown in Figure 7B. It achieved high quality spike and LFP recordings for approximately 3 weeks. In a second animal to evaluate our embedded microdrives composed of 3D printed components and standard fasteners, we implanted 2 hippocampal probes, 1 affixed to a metal micro-drive, and the other affixed to our embedded microdrive, as shown in Figure 7C. We recorded high-quality LFP signal from both hippocampal probes for greater than 4 weeks, as highlighted in Figure 7D by the presence of signature delta (0.5–4 Hz) and spindle waves (11–16 Hz) during sleep. The ability of our headcap to accommodate both metal microdrives and embedded 3D-printed microdrives while obtaining stable recordings over multiple weeks indicates the interface plate is well-designed to accurately target typical recording regions while achieving signal comparable to standard surgeries. Further, the embedded 3D printed drive on the headcap is the same size as commercially available drives and offers the advantage of pre-mounting the probe to the headcap with pre-calibrated stereotaxic coordinates prior to surgery, eliminating the need for iterative implantations and excessive handling of individual probes during multi-implant procedures. Also, our 3D printed embedded microdrives are highly customizable and reconfigurable (thanks to the digital design and 3D printing nature) and of much lower cost as compared to commercial metal counterparts.

### 3.3 Validation of THEM implantation system for chronic recordings

**Figure 8:**
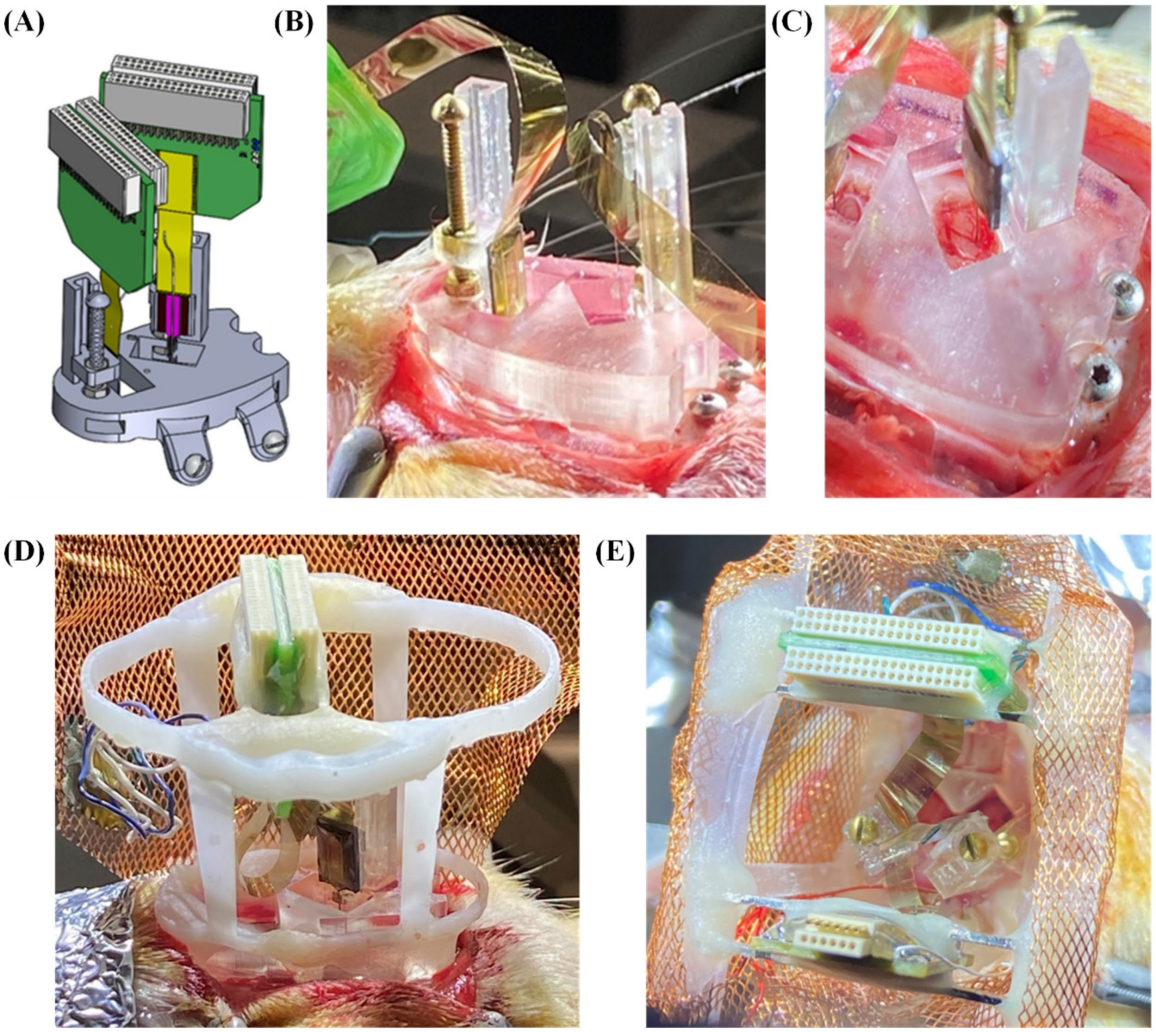
Surgical testing and validation of the THEM system with variations in upper framework: **(A)** schematic view of double insertion using THEM system without upper framework; **(B)** double probe implantation without pre-attached upper framework, allowing good visualization of probe insertions from multiple angles; **(C)** overhead view of CA1 insertion emphasizing ability to visualize brain penetration; **(D)** double implantation with pre-attached upper framework; **(E)** double implantation using fully assembled THEM system with upper framework pre-wrapped in copper mesh.

For the remainder of our trials, we recorded local field potentials (LFP) and single unit spiking activity over the course of many weeks from probes mounted to our embedded microdrives only (Figure 8A; *n* = 10 including 4 single implant, 6 double implants). These had variations in the upper framework to determine both the feasibility of the THEM system for quality chronic recordings as well as ease of implantation when using modified upper versions of the headcap. The three variations we tested were: (1) upper framework added during surgery (Figure 8B), allowing full visualization of insertions from the side and top (Figure 8C), (2) upper framework pre-mounted to interface plate without copper mesh (Figure 8D), allowing good visualization but limited access inside cap, and (3) fully assembled cap with copper mesh pre-attached and all probe reference wires soldered to mesh (Figure 8E). Using a partially completed upper framework as in Figure 8D offers the benefit of easier access to cranial windows as well as better visualization of implantation, and though the cap is not fully complete at the time of insertions, surgeries are able to be completed much faster than traditional methods. Alternatively, using a fully pre-assembled cap with copper mesh (as in Figure 8D) markedly improves surgical time and efficiency (to be elaborated in section 3.4), though requires some skill and attention when filling the craniotomy windows post-implantation. We achieved both efficient and repeatable surgeries yielding high-quality recordings with a fully pre-assembled rectangular version of the upper headcap design as shown in Figures 4, 8E and we recommend such upper support method for future implementations of the THEM system. Besides the specific implantation coordinates in this study, we suspect this design will also provide the greatest benefit to most researchers due to the simple framework structure that allows both spacing flexibility and good implantation visualization for most brain targets.

**Figure 9:**
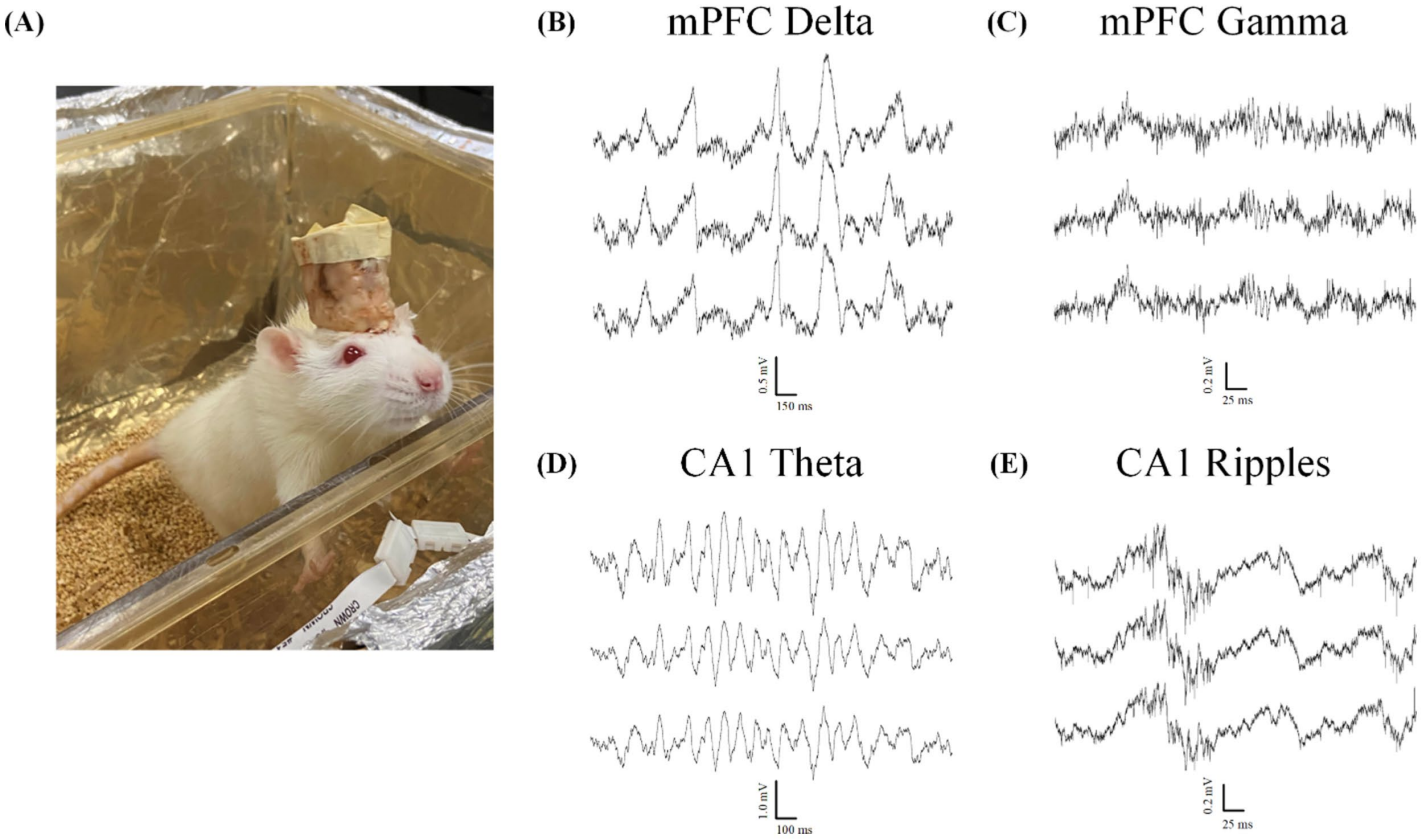
Local field potential recordings indicate normal physiology during wake and sleep. All data taken from animals with two probes inserted through the headcap using the proposed embedded microdrives (*n* = 6). **(A)** Example Sprague–Dawley rat in home-cage recording chamber. **(B–E)** Example electrophysiology recordings from two silicon probes implanted with our headcap system showing 3 representative traces from a 128-channel data set highlighting. **(B)** Delta oscillations (0.5–4 Hz) characteristic of normal NREM sleep rhythms. **(C)** Gamma oscillations (40–100 Hz) during the active wake state. **(D)**Theta rhythms (5–12 Hz) and **(E)** ripples (140–200 Hz) seen during both active exploration and REM sleep states.

Animals were recorded once per week during home-cage sessions lasting 2–6 h, as in Figure 9A. All 10 animals displayed markers of quality LFP recordings lasting at least 4 weeks, while 7 animals displayed quality LFP for over 6 weeks. Assessment of LFP was determined by presence of known spectral frequency power during particular sleep and wake states. All mPFC probes were able to reliably capture the presence of delta oscillations (0.5–4 Hz) during non-rapid-eye-movement sleep (NREM; Figure 9B) and gamma oscillations (40–100 Hz; Figure 9C) during active wake states. We also reliably recorded strong theta oscillations (5–12 Hz; Figure 9D) as well as ripples (140–200 Hz; Figure 9E) in our hippocampal probes during both rapid-eye-movement (REM) sleep and active exploration, indicating normal physiology and quality cortical LFP recordings.

**Figure 10:**
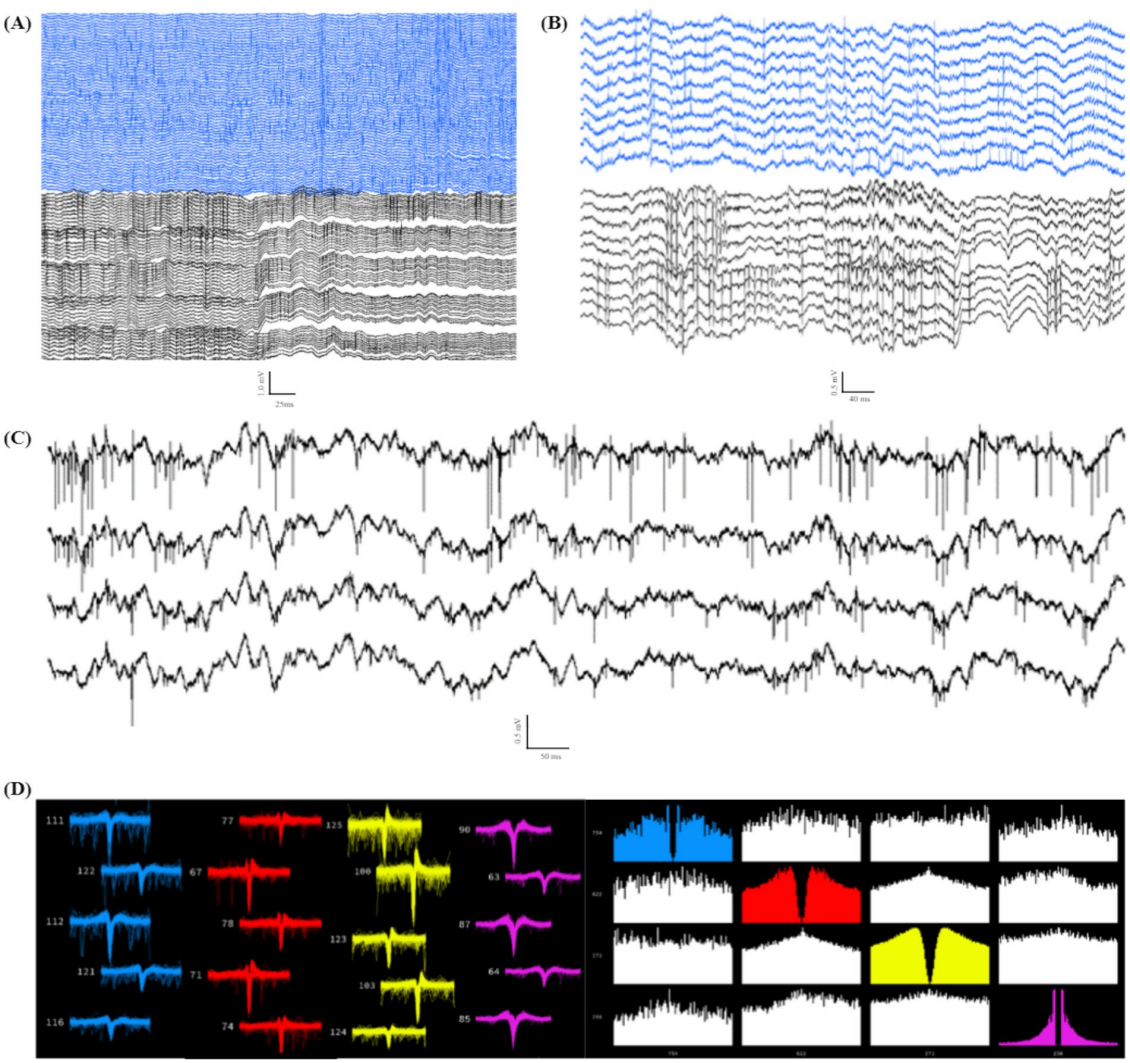
Individual unit spiking data from double implant headcap animals indicating high-quality recordings capable of capturing spiking data. **(A)** Example data of spiking in 128 channels (PFC in blue, CA1 in black). **(B)** Example subset of channels from **(A)**, highlighting spiking activity from both recorded regions of a single headcap animal. **(C)** Four example traces from **(B)** focusing on high-resolution spiking in individual channels. **(D)** Example showing 4 distinct sorted units (Kilosort2) (left: waveform view indicating high-quality spike waveforms; right: correlogram view demonstrating distinct firing patterns from the same 4 units shown on the left).

A major advantage of using high-channel-density probe implantations compared to other less-invasive techniques (i.e., EEG, calcium imaging) is the ability to detect single unit spiking activity on fast timescales across many electrodes. Because of the invasive nature of probe implantations, acquiring spiking signals from individual neurons requires smooth and stable insertions to limit swelling of and damage to the brain tissue. After refining our drive technology for stable implantation, we observed and recorded high-quality spiking data from many channels simultaneously (average 26.3 ± 8.6 units per probe ± SEM, *n* = 4; Figure 10), indicating mechanical stability and insertion control with limited tissue damage and swelling. We also observed spiking activity for chronic recordings, as many of our animals maintained quality spiking activity over the course of multiple weeks. The results indicate that our THEM system is capable of recording brain signals in a manner comparable with conventional surgery methods while markedly improving implantation efficiency and repeatability. Spikes were detected in 10 out of 10 animals in recordings within the first week after surgery. Spiking at 3 weeks was less reliable, and was seen in 7 out of 10 recordings, while only 3 animals displayed spiking beyond 4 weeks.

### 3.4 Evaluation of surgical time savings

The THEM system simplifies the neural probe implantation surgery and reduces surgical time in two ways. First, with the neural probes being pre-mounted on corresponding pre-assembled microdrives embedded in the 3D printed headcap, the THEM system eliminates the iterative and time-consuming steps including handling each of the probes, loading onto guidance arms, calibrating targeting through stereotaxic coordinate adjustments, inserting, anchoring, removing the guidance arm, and restarting the process for each additional probe. Based on our surgeries in this study, implanting each neural probe without the THEM system pre-assembled microdrives took about 29 min (*n* = 5), which decreased by over 75% to 7 min per probe (*n* = 9) with the THEM system. The total temporal difference is greater the more probes are implanted. Moreover, the 3D-printed upper support structure in the THEM system also dramatically reduced the time needed for manual construction of the upper framework. In this study, the average time between the last probe insertion and the surgery end without THEM system was 146 min (*n* = 4). In comparison, by using the pre-assembled upper support structure (Figure 4E), the time was decreased by 58% to 62 min (*n* = 3). Such difference would likely be extended in a non-linear manner for surgeries involving more than two probes due to the difficulty of managing multiple connectors in an increasingly small space. Thus, as the number of probes implanted increases, having a pre-assembled headcap with embedded microdrives decreases the surgical time by hours and dramatically decreases surgical burden, resulting in more efficient and repeatable implantations while opening the possibility of implanting many more probes than is currently reasonable.

## 4 Discussion

The current study presents a unique 3D printed headcap with embedded microdrive (THEM) system that removes the need for heavy stereotaxic use and allows attachment of pre-assembled cap-probe systems to the animal head. Through iterative design and prototyping, we encountered and addressed several challenges including system stability, weight distribution, targeting accuracy, and fabrication complexities. Our new headcap system also addresses burdens that arise during typical probe implantation surgeries, such as the need for manual alignment, probe/drive anchoring, and housing fabrication within a small space over hours-long surgeries.

During conventional implantation surgeries, a stereotaxic arm is used to guide insertion, after which the probe must be anchored to the skull prior to disconnecting the arm. This critical point during standard surgery risks vibration and movement damage to brain tissue when disconnecting the stereotaxic arm, and risks probe damage or contamination while anchoring the probe/drive to the skull within a small space. When the THEM system is employed, much of the surgical work is shifted to pre-surgical preparation—which is more controllable and less pressured since no animal is under anesthesia and the cap can be easily rotated and manipulated as needed. The developed system uses drives and a bregma-aligned computer-aided design (CAD) to allow users to assemble stereotaxically specific implants before the surgery day to reduce surgery time and difficulty. Once the headcap is anchored to the skull, each probe is already accurately positioned above a bregma-aligned craniotomy window and can be lowered to the desired target depth by turning the drive screws. Surgical efficiency is particularly improved for higher implant numbers since the components of the cap are preassembled prior to surgery. Also, the pre-printed bregma-referenced drive system allows for fine adjustments of insertion depth, allowing for post-surgical targeting of spiking populations. This improved surgical efficiency also reduces the amount of time the animal is under anesthesia and decreases risk of surgical complications due to anesthetic length/depth and reliance on surgical performance in small spaces.

The THEM system also incorporates a rectangular-shaped fully pre-assembled upper framework designed specifically to better manage multiple probes for more efficient, successful, and repeatable insertions. In standard probe surgeries, the probe packaging (connector/amplifier) must be managed with care (put aside, allowed to dangle, or held un-stably) while the recording ends of the probes are being inserted. The stereotaxic arm is then re-loaded with the next probe, and the process is repeated, with increased difficulty due to decreased workspace, and increased wire and probe packaging management for each additional probe. Our inexpensive and lightweight system allows the surgeon to pre-mount the probes/drives on the interface plate and organize, support, and protect the probe packaging with the upper cap prior to surgery. This approach simplifies the implantation process, and avoids peri-surgical handling and anchoring of each probe, thereby limiting the risk of probe damage, brain damage, and contamination. We successfully used this cap for 12 animals with either 1 or 2 probe implants, resulting in more efficient, faster, and easier surgeries. After our trial surgeries, based on remaining available space, we estimate the cap could accommodate up to 3 probes per hemisphere, or 6 total. Headstage spacing and the weight of the headcap are the limiting factor for implanting greater than 4 probes, though further testing will be required to determine an upper limit. Testing the full limits of the system is outside the scope if this development report, but many potentially ambitious experiments are currently limited by traditional manual surgical methods.

Our headcap system is also versatile and customizable. First, the drives are compatible with a variety of available probes that can be affixed to the embedded microdrives during surgical preparation. The locations of the craniotomy windows and brain targets are easily customizable due to the modular digital design. Pre-planned targeting is highly accurate since the 3D printed headcap is aligned to a universal bregma coordinate with minimal error (A/P axis standard error of the mean = 0.25 mm, M/L axis standard error of the mean = 0.07 mm, *n* = 6) (Yi et al., 2022). Having a modular system for accurate targeting as well as management of the hardware and packaging with the upper support provides more opportunity for customization to accommodate other implantation tools including optogenetics, calcium imaging windows, electrode wires/bundles, various types of MEAs, fiber photometry, or others. Since the upper support is a separate piece designed to be attached to the interface plate, it can be assembled either prior to surgery or post-implantation or could be easily modified to accommodate various framework requirements. With the THEM system shown above, a full two-probe headcap weighs less than 10 g, minimizing its impact on the rodent subjects and making it ideal for chronic recordings. Further, because the 3D components are lightweight and easily modifiable, our system could be easily adapted to create the ideal design for other recording approaches. Besides neural probes, the THEM system could also be used for microwire-based MEAs as in our previously published work (Yi et al., 2022). Given the custom digital design and highly reconfigurable nature of the THEM platform, it could also potentially serve as a general platform and be coupled with other developed microdrives (Voigts et al., 2013; Kim et al., 2020) and insertion actuators (Smith et al., 2022) for all types of MEAs and optical fibers.

With the advancement of probe technology including the increased number of electrode sites, customization of design and spacing, and integration of the amplifier chips into the probe connector, probes are becoming more powerful, but are also more costly, making recovery more desirable. Typical implantation procedures limit recoverability by relying on cement and adhesives to fully encapsulate the probes but such practice can make it difficult to recover the probe for reuse. Our modular headcap design improves the post-experiment probe recovery process because the probe is well-anchored to, and protected by, the headcap. Minimal cement is needed during the implantation process and is used mainly for anchoring the headcap to the skull, a safe distance from the probes. For recovery, the probes can be raised from the brain using the drive screws, and the entire headcap assembly removed from the skull. Because the headcap is made from inexpensive resin, it is easily cut-away to give access to the drive assembly for easier recovery.

Limitations include that our system was specifically designed for the skulls of Sprague–Dawley rats, and further work will need to be done to test if this headcap can be adapted to other rat strains. Also, we have had relative difficulty recording spikes for more than 3 weeks consistently. This is not a problem for most applications, but a few experimentalists might desire multi-month recordings. Further testing and development would be required to solve this problem with one candidate mechanism being sub-micron instability slowly reducing tissue quality. This could originate from an unstable attachment of the cap to the skull or from vibration initiating at the top of the drives. Additionally, though we are optimized thus far for vertical non-angled insertions, we are not aware of reasons why this system could not support angled insertions if either printed separately and used in conjunction with a stereotaxic frame or angled and printed atop the interface plate. Finally, future work will be required to determine the maximum number of probes that can be implanted at once. However, with the evolution in the design of probe packaging that continues to evolve, a CAD-based system such as ours can be easily modified to match those changes and needs.

## 5 Conclusion

Our study presents a significant advancement in rodent neuroengineering by demonstrating the practicality and efficiency of a three-dimensional-printed headcap with an embedded microdrive (THEM) system for customizable multi-region brain recordings with neural probes. The novel integration of CT-based digital headcap design with custom insertion targets, embedded customizable microdrives, and rapid prototyping through 3D printing offers a streamlined, less invasive approach to neural recordings, setting a new standard for surgical precision and simplicity in the study of brain function.

## Data Availability

The datasets presented in this study can be found in online repositories. The names of the repository/repositories and accession number(s) can be found at: https://github.com/LeiChenLab/3D_Printed_Skull_Cap.

## References

[ref1] Allen W. E. Chen M. Z. Pichamoorthy N. Tien R. H. Pachitariu M. Luo L.. (2019). Thirst regulates motivated behavior through modulation of brainwide neural population dynamics. Science 364:253. doi: 10.1126/science.aav3932 30948440 PMC6711472

[ref2] Bimbard C. Takács F. Fabre J. Melin M. O’Neill N. Robacha M.. (2023) Reusable, flexible, and lightweight chronic implants for Neuropixels probes. *bioRxiv*. Available at: 10.1101/2023.08.03.551752. [Epub ahead of preprint]

[ref3] Coyle S. M. Ward T. E. Markham C. M. (2007). Brain–computer interface using a simplified functional near-infrared spectroscopy system. J. Neural Eng. 4, 219–226. doi: 10.1088/1741-2560/4/3/007, PMID: 17873424

[ref4] Durand S. Heller G. R. Ramirez T. K. Luviano J. A. Williford A. Sullivan D. T.. (2023). Acute head-fixed recordings in awake mice with multiple Neuropixels probes. Nat. Protoc. 18, 424–457. doi: 10.1038/s41596-022-00768-6, PMID: 36477710

[ref5] Frank J. A. Antonini M. J. Anikeeva P. (2019). Next-generation interfaces for studying neural function. Nat. Biotechnol. 37, 1013–1023. doi: 10.1038/s41587-019-0198-8, PMID: 31406326 PMC7243676

[ref6] Headley D. B. DeLucca M. V. Haufler D. Paré D. (2015). Incorporating 3D-printing technology in the design of head-caps and electrode drives for recording neurons in multiple brain regions. J. Neurophysiol. 113, 2721–2732. doi: 10.1152/jn.00955.2014, PMID: 25652930 PMC4416572

[ref7] Hong K. S. Ghafoor U. Khan M. J. (2020). Brain–machine interfaces using functional near-infrared spectroscopy: a review. Artif. Life Robot. 25, 204–218. doi: 10.1007/s10015-020-00592-9

[ref8] Hong G. Lieber C. M. (2019). Novel electrode technologies for neural recordings. Nat. Rev. Neurosci. 20, 330–345. doi: 10.1038/s41583-019-0140-6, PMID: 30833706 PMC6531316

[ref9] Juavinett A. L. Bekheet G. Churchland A. K. (2019). Chronically implanted Neuropixels probes enable high-yield recordings in freely moving mice. eLife 8:e47188. doi: 10.7554/eLife.47188, PMID: 31411559 PMC6707768

[ref10] Jun J. J. Steinmetz N. A. Siegle J. H. Denman D. J. Bauza M. Barbarits B.. (2017). Fully integrated silicon probes for high-density recording of neural activity. Nature 551, 232–236. doi: 10.1038/nature24636, PMID: 29120427 PMC5955206

[ref11] Juska V. B. Maxwell G. Estrela P. Pemble M. E. O’Riordan A. (2023). Silicon microfabrication technologies for biology integrated advance devices and interfaces. Biosens. Bioelectron. 237:115503. doi: 10.1016/j.bios.2023.115503 37481868

[ref12] Khodagholy D. Gelinas J. N. Thesen T. Doyle W. Devinsky O. Malliaras G. G.. (2015). NeuroGrid: recording action potentials from the surface of the brain. Nat. Neurosci. 18, 310–315. doi: 10.1038/nn.3905, PMID: 25531570 PMC4308485

[ref13] Kim H. Brünner H. S. Carlén M. (2020). The DMCdrive: practical 3D-printable micro-drive system for reliable chronic multi-tetrode recording and optogenetic application in freely behaving rodents. Sci. Rep. 10:11838. doi: 10.1038/s41598-020-68783-9, PMID: 32678238 PMC7366717

[ref14] Luo T. Z. Bondy A. G. Gupta D. Elliott V. A. Kopec C. D. Brody C. D. (2020). An approach for long-term, multi-probe Neuropixels recordings in unrestrained rats. eLife 9:e59716. doi: 10.7554/eLife.59716, PMID: 33089778 PMC7721443

[ref15] Melin M. D. Churchland A. K. Couto J. (2023). Large scale, simultaneous chronic neural recordings from multiple brain areas. *bioRxiv*. Available at: 10.1101/2023.12.22.572441. [Epub ahead of preprint]

[ref16] Min B. K. Marzelli M. J. Yoo S. S. (2010). Neuroimaging-based approaches in the brain–computer interface. Trends Biotechnol. 28, 552–560. doi: 10.1016/j.tibtech.2010.08.002, PMID: 20810180

[ref17] Mizuta K. Sato M. (2024). Multiphoton imaging of hippocampal neural circuits: techniques and biological insights into region-, cell-type-, and pathway-specific functions. Neurophotonics. 11:033406. doi: 10.1117/1.NPh.11.3.033406, PMID: 38464393 PMC10923542

[ref18] Paxinos G. Watson C. (2006). The rat brain in stereotaxic coordinates. *Sixth edition*, Cambridge: Academic Press.

[ref19] Shen K. Chen O. Edmunds J. L. Piech D. K. Maharbiz M. M. (2023). Translational opportunities and challenges of invasive electrodes for neural interfaces. Nat. Biomed. Eng. 7, 424–442. doi: 10.1038/s41551-023-01021-5, PMID: 37081142

[ref20] Smith R. D. Kolb I. Tanaka S. Lee A. K. Harris T. D. Barbic M. (2022). Robotic multi-probe single-actuator inchworm neural microdrive. eLife 11:e71876. doi: 10.7554/eLife.71876, PMID: 36355598 PMC9651949

[ref21] Stieglitz T. (2020). Of man and mice: translational research in neurotechnology. Neuron 105, 12–15. doi: 10.1016/j.neuron.2019.11.030 31951526

[ref22] van Daal R. J. Aydin Ç. Michon F. Aarts A. A. Kraft M. Kloosterman F.. (2021). Implantation of Neuropixels probes for chronic recording of neuronal activity in freely behaving mice and rats. Nat. Protoc. 16, 3322–3347. doi: 10.1038/s41596-021-00539-9 34108732

[ref23] Vandecasteele M. Royer S. Belluscio M. Berényi A. Diba K. Fujisawa S.. (2012). Large-scale recording of neurons by movable silicon probes in behaving rodents. J. Vis. Exp. 61:e3568. doi: 10.3791/3568 PMC3399468 22415550

[ref24] Viventi J. Kim D. H. Vigeland L. Frechette E. S. Blanco J. A. Kim Y. S.. (2011). Flexible, foldable, actively multiplexed, high-density electrode array for mapping brain activity *in vivo*. Nat. Neurosci. 14, 1599–1605. doi: 10.1038/nn.2973, PMID: 22081157 PMC3235709

[ref25] Voigts J. Siegle J. H. Pritchett D. L. Moore C. I. (2013). The flexDrive: an ultra-light implant for optical control and highly parallel chronic recording of neuronal ensembles in freely moving mice. Front. Syst. Neurosci. 7:8. doi: 10.3389/fnsys.2013.00008 23717267 PMC3652307

[ref26] Vöröslakos M. Petersen P. C. Vöröslakos B. Buzsáki G. (2021). Metal microdrive and head cap system for silicon probe recovery in freely moving rodent. eLife 10:e65859. doi: 10.7554/eLife.65859, PMID: 34009122 PMC8177890

[ref27] Won S. M. Song E. Reeder J. T. Rogers J. A. (2020). Emerging modalities and implantable technologies for neuromodulation. Cell 181, 115–135. doi: 10.1016/j.cell.2020.02.054, PMID: 32220309

[ref28] Yi D. Hartner J. P. Ung B. S. Zhu H. L. Watson B. O. Chen L. (2022). 3D printed skull cap and benchtop fabricated microwire-based microelectrode array for custom rat brain recordings. Bioengineering 9:550. doi: 10.3390/bioengineering9100550, PMID: 36290518 PMC9598465

